# Advances in the multimodal management of central nervous system solitary fibrous tumors

**DOI:** 10.3389/fonc.2026.1778689

**Published:** 2026-07-06

**Authors:** Zhongxin Yang, Xun Xia

**Affiliations:** 1 Department of Neurosurgery, School of Clinical Medicine, Chengdu Medical College, Chengdu, Sichuan, China; 2 Department of Neurosurgery, The First Affiliated Hospital of Chengdu Medical College, Chengdu, Sichuan, China; 3 Key Laboratory of Neural Injury and Repair, Chengdu Medical College of Sichuan Province, Chengdu, Sichuan, China

**Keywords:** anti-angiogenic therapy, central nervous system (CNS), chemotherapy, comprehensive therapy, NAB2-STAT6 fusion gene, radiotherapy, solitary fibrous tumor (SFT)

## Abstract

Solitary fibrous tumors (SFTs) of the central nervous system (CNS) are rare mesenchymal fibroblastic neoplasms. Although surgical resection remains the primary treatment, achieving gross-total resection is often challenging due to the frequent proximity of these tumors to critical neurovascular structures. Consequently, postoperative recurrence rates can be as high as 43%, and there is a notable risk of metastasis, underscoring the need for effective multimodal management.This review synthesizes current evidence on therapeutic strategies for CNS SFTs.Maximal safe surgical resection remains the cornerstone of local control. For patients undergoing subtotal resection, or those with high-risk pathological features (e.g., WHO grade II/III, high mitotic count, or necrosis), postoperative radiotherapy can significantly improve local control rates.Regarding systemic therapy, anti-angiogenic agents (e.g., pazopanib) have demonstrated superior disease control compared to conventional chemotherapy in patients with advanced, recurrent, or metastatic non-dedifferentiated SFT. Cytotoxic chemotherapy (e.g., doxorubicin) is now largely reserved for refractory or dedifferentiated subtypes. Immunotherapy (e.g., PD-1/PD-L1 inhibitors) has shown limited but promising activity in select patients, with PRAME emerging as a potential novel immunotherapeutic target.Optimal patient management requires a multidisciplinary team (MDT) approach involving specialists from neurosurgery, radiation oncology, and medical oncology.Treatment decisions should be individualized based on the extent of resection, tumor grade, location, and patient-specific factors. Lifelong, regular MRI surveillance is crucial for early detection of recurrence.Future directions should focus on conducting prospective clinical trials to generate higher-level evidence, further elucidating the NAB2-STAT6 fusion oncogene pathway, and developing rational combination therapies to improve outcomes for patients with this challenging tumor.

## 1 Introduction

Solitary fibrous tumor (SFT) is a rare neoplasm originating from mesenchymal fibroblastic cells. The first case of pleura-originated SFT was reported by Klemperer et al. in 1931 (1). In cases of SFT involving central nervous system (CNS), surgical resection remains the primary treatment modality. Gross total resection significantly reduces the risk of recurrence and improves prognosis, whereas residual tumor is associated with a higher risk of recurrence and malignant transformation (2, 3). Nevertheless, recurrence has been reported even after gross-total resection, which may be attributed to the tumor’s malignant potential, rich vascularity, or indistinct borders with neural tissue (4). The recurrence rate following initial surgery can be as high as 43%, with recurrence intervals ranging from 1 to 25 years postoperatively and a mean recurrence time of approximately 5.8 years (5). For intracranial meningeal SFTs, the recurrence rate is at least 37% (mean 4.7 years), with a symptomatic metastasis rate of 10% (6). In the spine, SFTs have a recurrence rate of approximately 43% (mean 5.8 years) and a metastasis rate ranging from 11% to 25% (7, 8). Multiple recurrences are not uncommon. In a single-center study by Gou et al., among 42 SFT patients, 22 (52%) experienced recurrence, including 13 (31%) with one recurrence, 4 (10%) with two recurrences, and 5 (12%) with three or more recurrences (9).

**Figure 1:**
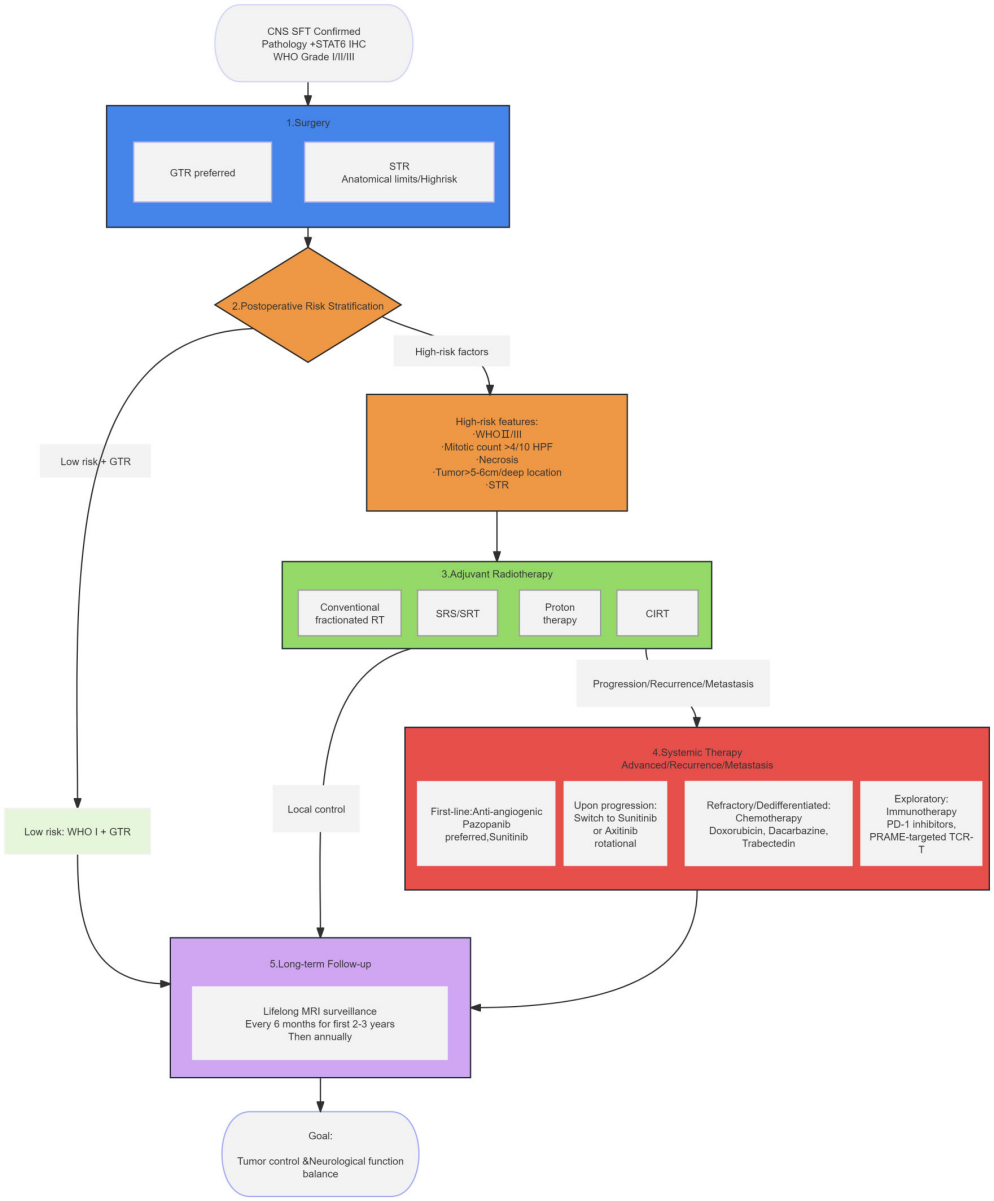
Multimodal management algorithm for central nervous system solitary fibrous tumors (CNS SFT). This flowchart outlines the recommended stepwise approach to CNS SFT treatment. After histopathological confirmation (including STAT6 immunohistochemistry) and WHO grading, maximal safe surgical resection is pursued. Gross-total resection (GTR) is preferred, but subtotal resection (STR) may be required due to anatomical constraints. Postoperatively, patients are stratified by risk of recurrence. High-risk features include WHO grade II/III, mitotic count >4/10 high-power fields (HPF), tumor necrosis, large tumor diameter (>5–6 cm), deep/skull base location, and STR. For high-risk or STR patients, adjuvant radiotherapy is recommended, with options including conventional fractionated radiotherapy, stereotactic radiosurgery (SRS), proton therapy, or carbon ion radiotherapy (CIRT). For advanced, recurrent, or metastatic disease, systemic therapy is indicated: first-line anti-angiogenic therapy (pazopanib as preferred agent, with rotational options sunitinib or axitinib upon progression); for refractory or dedifferentiated subtypes, chemotherapy (doxorubicin, dacarbazine, trabectedin) is used; immunotherapy (e.g., PD-1 inhibitors, PRAME-targeted TCR-T) remains exploratory. Lifelong regular MRI surveillance (every 6 months for the first 2–3 years, then annually) is essential for all patients. The ultimate goal is to achieve an optimal balance between tumor control and preservation of neurological function. CNS, central nervous system;SFT, solitary fibrous tumors; GTR, gross-total resection; STR, subtotal resection; SRS, stereotactic radiosurgery; SRT, stereotactic radiotherapy;CIRT, carbon ion radiotherapy; PD−1, programmed cell death protein 1; PRAME, preferentially expressed antigen in melanoma; TCR−T, T−cell receptor−engineered T cell.

Given the high postoperative recurrence rate and significant risk of metastasis in CNS SFT, comprehensive postoperative management is crucial. Current postoperative treatment options include radiotherapy (such as stereotactic radiosurgery, proton therapy, and carbon ion therapy), chemotherapy, anti-angiogenic therapy, and immunotherapy. However, the overall efficacy of these treatments remains limited due to therapy-related toxicities (e.g., side effects of chemotherapy or neurological injury following radiotherapy) and ongoing controversies regarding the effectiveness of some modalities. Furthermore, because SFT is a rare disease, most available studies are limited to case reports or small retrospective series, and there is a lack of large-scale, prospective clinical trial data. This article aims to systematically review the latest advances in comprehensive postoperative treatments for CNS SFT, with the goal of providing a guidance for reducing postoperative recurrence and metastasis, and optimizing treatment strategies. Meanwhile, the proposed management algorithm is summarized in **Figure 1**.

## 2 Epidemiology and clinical features

SFTs can occur in various locations throughout the body but most commonly arise in the pleura. Approximately 20%–30% of SFTs originate in the CNS (8). CNS SFTs are rare, accounting for 0.4% of all primary CNS tumors and less than 1% of intracranial tumors. Spinal SFTs are even rarer, with an incidence ratio of approximately 1:10 compared to cranial SFTs (10, 11). The thoracic spinal cord is the most commonly affected vertebral region (38.3%), followed by the cervical (34.6%) and lumbar (24.8%) regions, while lumbosacral SFTs are the rarest (1.5%) (12, 13). SFTs predominantly occur in the intradural extramedullary space, although intramedullary spinal SFTs have also been reported and appeared to be more frequent than intraparenchymal brain SFTs (14). Spinal SFTs are generally benign, with malignant cases being extremely rare, although such instances have been documented in the cervical, thoracic, and lumbar regions (5, 15).

**Figure 2:**
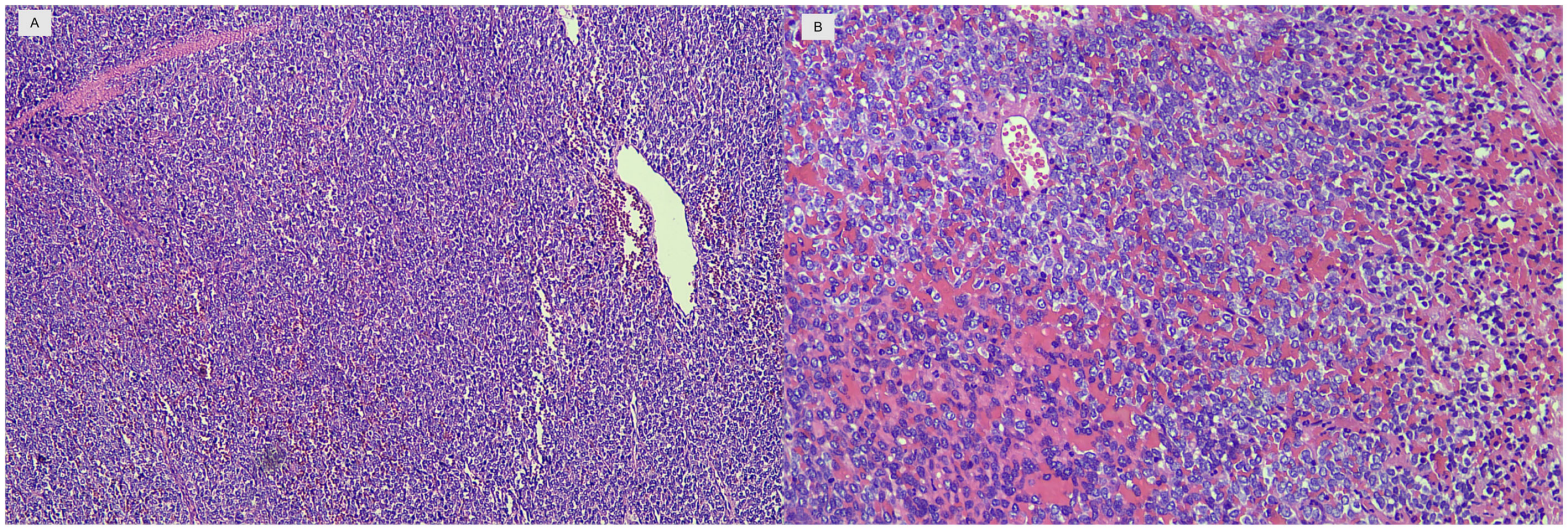
Foramen magnum SFT(WHO II) hematoxylin and eosin (H&E) (x200). This pathological is from a 35-year-old male patient in our institution who experienced a second recurrence after surgery for a foramen magnum tumor (September 28, 2025). H&E: The tumor cells were spindle-shaped and oval, arranged in a disorganized, non-specific pattern, occasionally forming fascicular, interlacing, storiform, or herringbone architectures. Hypocellular and hypercellular areas alternated within the tumor. Dilated blood vessels with frequent marked hyalinization of the vessel walls were observed **(A)**. The tumor cells had ill-defined borders, uniform nuclear chromatin, and no obvious atypia. Approximately 5 mitotic figures per 10 high-power fields (5/10 HPF) were identified, and no tumor necrosis was seen **(B)**. HPF, high-power fields;H&E, hematoxylin and eosin.

**Figure 3:**
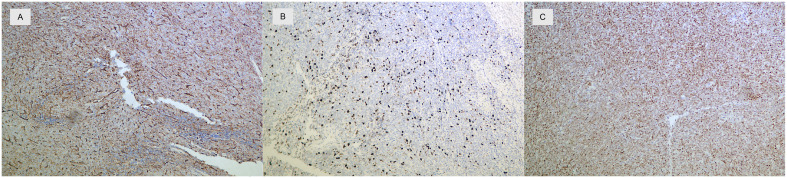
Foramen magnum SFT(WHO II) Immunohistochemistry (IHC)(x 200. This pathological result is from a 28-year-old male patient in our center who experienced a second recurrence after surgery for a foramen magnum tumor (September 28, 2025). IHC: The tumor cells expressed CD34 (+) **(A)**, Ki-67 (10%+) **(B)**, and STAT6 (+) **(C)**. IHC, immunohistochemistry;CD34, cluster of differentiation 34;Ki-67, Ki-67 Antigen;STAT6, Signal Transducer and Activator of Transcription 6.

According to the 2016 WHO classification, most SFTs are histologically benign, with only 10%–20% exhibiting borderline or malignant features (16). The 2021 WHO classification of CNS tumors introduced a revised, three-tier grading system (WHO grades I–III) based on mitotic count and necrosis. This update aims to provide greater grading flexibility, better align CNS SFTs with soft tissue tumor pathology standards, and reinforce the biological relationships among different tumor categories (17).Its representative pathological features are shown in **Figures 2**, **3**. The overall age-adjusted incidence of SFT is 3.77 per 10 million, and 23%–51% of tumors ultimately experience local recurrence or metastasis after initial treatment (18, 19).

## 3 General principles of management

Patients with CNS SFT should be managed at specialized sarcoma treatment centers by a multidisciplinary team, including pathologists, radiologists, neurosurgeons, radiation oncologists, and medical oncologists experienced in this disease, in order to develop individualized treatment plans (20). To assess treatment efficacy, the combined use of the Response Evaluation Criteria in Solid Tumors (RECIST) and Choi criteria—which defined as a ≥10% reduction in maximal tumor diameter or a ≥15% decrease in CT tumor density—may provide a more accurate evaluation, particularly for anti-angiogenic agents (21).

## 4 Surgical treatment

Gross-total resection is the cornerstone of CNS SFT treatment and is crucial for achieving long-term local control, as well as for reducing the risk of recurrence and metastasis (2, 3). Intracranial SFTs most commonly attach to the dura mater, with the highest prevalence at the tentorium cerebelli, followed by the frontal convexity, cerebellopontine angle, ventricles, falx cerebri, and posterior fossa (22). Prognosis after gross-total resection is significantly better than after subtotal resection. Although gross-total resection is achievable in many cases, some tumors are only amenable to partial resection due to their location, the frequent inability to assess intraoperative microscopic margins, and a tendency for fragmentation. Moreover, compared to SFTs in other locations, meningeal SFTs often exhibit higher cellular density and mitotic counts, which may contribute to a relatively poorer prognosis (23).Postoperative risk stratification and adjuvant treatment decision-making follow the steps outlined in **Figure 1**.

## 5 Radiotherapy

The value of adjuvant radiotherapy in the treatment of SFT has long been debated. Multiple retrospective studies suggest that although adjuvant radiotherapy may improve local control rates in both meningeal and non-meningeal SFTs, however, it does not appear to be effective in preventing neurological or systemic metastases (24–26). In the largest patient cohort to date (n=747), Sakhr Alshwayyat and colleagues found that postoperative adjuvant radiotherapy did not significantly lower mortality rates but was associated with improved overall survival (27). Similarly, Kinslow et al. found that radiotherapy combined with subtotal resection was associated with prolonged survival and offered additional benefit compared to subtotal resection alone (18).A multicenter study by Cohen-Inbar et al. found that stereotactic radiosurgery (SRS) provides reasonable local control for postoperative SFT patients, with a low risk of adverse effects (28). Kung et al. suggested that tumor aggressiveness may predict treatment response, noting that both the extent of surgical resection and adjuvant radiotherapy were significantly associated with progression-free survival (PFS) in WHO grade II SFTs, but not in indolent WHO grade I tumors (29). Similarly, a study by Anthony et al. (30)observed a trend toward reduced disease recurrence or progression (P = 0.18) and prolonged overall survival (OS) (P = 0.15) with adjuvant radiotherapy. Additionally, more extensive surgical resection showed a trend toward improved OS (P = 0.19), although neither finding reached statistical significance.A large retrospective analysis of 549 SFT patients found that 428 (78%) underwent surgery alone, while 121 (22%) received surgery followed by postoperative radiotherapy. After propensity score matching, adjuvant radiotherapy was associated with a significantly improved local control rate (p = 0.012), though this did not translate into a significant benefit in OS (25). Other smaller case series have reached similar conclusions: radiotherapy improves local control but has limited impact on OS (31, 32). These results differ from earlier views suggesting SFTs are highly radiosensitive. However, a study involving 40 patients treated with radical radiotherapy (60 Gy) reported an overall response rate (ORR) of 67%, a 5-year local control rate of 81.3%, and a 5-year OS of 87.5% (33).Based on current evidence, postoperative adjuvant radiotherapy is valuable for improving local control, particularly in cases of subtotal resection or when positive margins are anticipated or confirmed, and especially for tumors with high proliferative indices (active mitosis). However, its absolute impact on survival prognosis remains uncertain and requires further confirmation through prospective studies.

### 5.1 Radiotherapy dose

Multiple studies suggest that doses of ≥50–60 Gy may be associated with improved local control. The study by Mike Ton et al. indicated a lower risk of recurrence and better outcomes with an initial radiotherapy dose of ≥60 Gy (34). Dufour et al. (34, 35) reported that administering 50–60 Gy in combination with surgery reduced the local recurrence rate to 12.5%. Similarly, Rutkowski et al. confirmed that doses exceeding 50 Gy improved PFS in WHO grade I SFT, with acceptable side effects (34, 36). However, the impact of radiotherapy on OS remains unclear. For example, Guthrie et al. found that adjuvant radiotherapy significantly prolonged PFS from 34 to 75 months, but did not demonstrate a clear improvement in OS, possibly due to the limited sample size (37).It is important to note that dose reduction may be necessary for tumors in special locations. Antonio Colamaria et al. (5) reported a case involving a patient with a cervical spinal canal WHO grade II SFT who received 24 Gy of adjuvant radiotherapy postoperatively and showed no tumor recurrence after two years of follow-up. Additionally, in 2021, Shanta Thapa et al. (38) reported a case of a pituitary region tumor treated with fractionated stereotactic radiosurgery (a marginal dose of 30 Gy delivered in five fractions) after subtotal resection, which achieved a favorable outcome.

### 5.2 High-precision radiotherapy techniques

Advances in radiotherapy have introduced precision techniques such as carbon ion radiotherapy, proton therapy, and stereotactic radiosurgery. These modalities provide new options for the treatment of CNS SFTs, especially for lesions located near critical neural structures, those that are difficult to resect completely via surgery, or in cases of recurrence.

#### 5.2.1 Particle radiotherapy: protons and carbon ions

For lesions adjacent to critical structures—such as the brainstem, spinal cord, or optic nerves—or for tumors resistant to conventional radiotherapy, particle radiotherapy (using protons or carbon ions) offers significant advantages due to the unique physical dose distribution of these particles, known as the Bragg peak.

##### 5.2.1.1 Carbon ion radiotherapy

Carbon ion radiotherapy (CIRT) has emerged as an important radical treatment for tumors that are unresectable or resistant to conventional radiotherapy (39). The core of CIRT advantages are twofold. First, CIRT provides a unique physical dose distribution characterized by the Bragg peak, which allows highly concentrated, high-dose radiation to be delivered precisely to the tumor target volume. This property is especially valuable for treating recurrent tumors located near critical structures such as the brainstem or spinal cord (40). Second, CIRT has a higher linear energy transfer, enabling it to destroy tumor cells more effectively while causing less radiation damage to the surrounding healthy tissues.This results in a higher relative biological effectiveness (41, 42). These characteristics make CIRT particularly promising for the treatment of radioresistant tumors.In recent years, case reports have demonstrated the clinical value of CIRT in treating CNS SFTs. The key clinical details of these cases are summarized in **Table 1**. While these preliminary results suggest that CIRT may provide effective local control for some refractory SFTs, its long-term efficacy and safety—particularly in relation to the CNS and spinal cord—still require confirmation through longer follow-up periods and larger-scale studies. Attention should also be given to potential late side effects such as radiation myelopathy, tissue atrophy, or vascular lesions (45).

**Table 1:** Summary of case reports on CIRT for CNS SFT.

Researcher (Year)	Case location	Treatment context	Prescribed dose & fractionation	Follow-up & outcome
Shiba et al. (2023) (43)	Cerebellopontine angle	Unresectable primary tumor	64 Gy/16 fractions	2 years: no recurrence, metastasis, or late toxicity
Tomomatsu et al. (44)	T4–5 spinal canal	Postoperative recurrence after 10 years; adjuvant after re-operation	64 Gy/16 fractions	2 years: reduced tumor volume, no radiation myelopathy
Murata et al. (42)	Thoracic spinal canal	Recurrent tumor	64 Gy/16 fractions	9 months: reduced tumor volume, no metastasis

In summary, owing to its precise dosimetry and favorable biological properties, CIRT provides a novel and intensified treatment strategy for challenging CNS SFTs. However, its optimal indications and long-term risk-benefit profile require further investigation.

##### 5.2.1.2 Proton therapy

Proton therapy takes advantage of the Bragg peak property of proton beams to deliver high-dose irradiation precisely to tumor targets while significantly reducing the dose to tissues located behind and surrounding the tumor (46). Compared to conventional photon radiotherapy, proton therapy offers a distinct advantage in dose distribution, which is especially beneficial for lesions adjacent to the optic pathway, brainstem, spinal cord, or critical developing brain regions in children. This advantage helps to increase tumor control rates, maximize the preservation of neurocognitive function, and reduce the risk of long-term complications (47). Preliminary clinical case reports (summarized in **Table 2**) support the use of proton therapy in the treatment of CNS SFTs. Given its physical and dosimetric advantages, proton therapy is particularly suitable for complex cases in which the tumor encases or is adjacent to critical neural structures, or in cases requiring re-irradiation, making it an important precision treatment option in the comprehensive management of SFTs.

**Table 2:** Comparison of high-precision radiotherapy techniques in CNS SFT.

Technology	Core principle/advantage	Optimal indications	Key clinical evidence
CIRT	1. Bragg peak: Offers precise dose distribution, enabling concentrated irradiation of the tumor target while sparing surrounding healthy tissue (40) 2. High relative biological effectiveness: High linear energy transfer that effectively kills radioresistant tumor cells (41, 42)	1. Tumors located near critical structures (e.g., brainstem, spinal cord) or those that are unresectable (43) 2.Recurrent tumors or those resistant to conventional radiotherapy (42, 44)	Case reports have demonstrated the use of CIRT for cerebellopontine angle and spinal SFT at a dose of 64 Gy/16 fractions, with follow-up showing favorable local control without severe toxicity (42–44) - Long-term side effects (e.g., radiation myelopathy, vascular lesions) require close monitoring (45)
Proton Therapy	Bragg peak: Provides precise dose distribution, significantly reducing radiation exposure to critical structures behind and around the tumor (e.g., optic pathway, brainstem, developing brain in children) (46, 47)	Tumors encasing or adjacent to critical neural structures (e.g., optic chiasm, brainstem) (48) 2. Cases requiring re-irradiation. 3. Patients with high priority for preservation of neurocognitive function (especially children) (47)	Case report: Case reports indicate proton therapy is effective in recurrent sellar SFT, using a boost dose of 66 Gy/30 fractions and a prophylactic dose of 54 Gy/30 fractions; short-term follow-up has shown good tumor control (48)
Technology	Core Principle /Advantage	Optimal Indications	Key Clinical Evidence
SRS/SRT	High precision and high dose: Delivers highly accurate, high-dose radiation to well-defined lesions in a single or few hypofractionated sessions (13, 28)	1. Small, well-defined residual or recurrent lesions (49, 50) 2. Lesions located away from critical organs. 3.Patients requiring a convenient, short treatment course	-Meta-analysis shows that postoperative SRS is effective and safe, with efficacy comparable to conventional fractionated radiotherapy and a lower incidence of acute radiation-induced brain injury (50). - For larger or multiply recurrent tumors, IMRT may be more suitable than SRS (9, 51)

#### 5.2.2 Stereotactic radiotherapy

Stereotactic radiosurgery (e.g., Gamma Knife) delivers highly precise, high-dose treatment to well-defined residual or recurrent lesions using either a single session or a few sessions of high-dose irradiation. Current evidence indicates that postoperative SRS is an effective and safe adjuvant treatment for intracranial SFT, with efficacy comparable to conventional fractionated radiotherapy but a lower incidence of acute radiation-induced brain injury. This highlights its value in achieving local control in both intracranial and spinal SFT. Both highly conformal fractionated radiotherapy and SRS can provide long-term disease control. However, for tumors with multiple recurrences or large volumes requiring better dose coverage, intensity-modulated radiotherapy may be more suitable than SRS (as shown in **Table 2**) (9, 13, 28, 49–51). The main advantages of SRS/SRT include convenience, short treatment duration, and suitability for small-volume lesions located away from critical organs.

### 5.3 Role and selection of radiotherapy in comprehensive treatment

High-precision adjuvant radiotherapy techniques play an essential role in the comprehensive management of CNS SFT. The final choice of radiotherapy technique should be based on multidisciplinary team discussions, with comprehensive consideration given to the specific characteristics and indications of different radiotherapy modalities, as well as tumor location, size, grade, proximity to critical structures, previous treatment history, and the patient’s overall condition, in order to achieve an optimal balance between tumor control and preservation of neurological function (**Table 2**).

## 6 Chemotherapy

Systemic chemotherapy plays a limited role in the treatment of CNS SFT and is generally not considered a first-line option. However, it remains a salvage strategy for patients with advanced disease, recurrence, refractory disease, or metastasis following the failure of standard treatments (52–54).

### 6.1 Traditional cytotoxic agents

Prospective evidence supporting the use of standard chemotherapy in SFT is extremely limited. Retrospective data indicate that anthracycline-based regimens (e.g., doxorubicin) yield an objective response rate of approximately 10.5%–20%, with a median PFS similar to that observed in other soft tissue sarcomas, at about 3–5 months (55). The efficacy of ifosfamide remains unclear. Although some studies suggest that the combination of doxorubicin and ifosfamide may prolong PFS (56), single-agent ifosfamide or ifosfamide-based regimens are associated with a median PFS of only 2–3 months and low response rates (about 10%) (55, 57).Dacarbazine (DTIC) and temozolomide have demonstrated promising activity. Preclinical studies in models of dedifferentiated SFT have shown that dacarbazine, either as monotherapy or in combination with doxorubicin, exerts anti-tumor effects by inducing DNA damage, and its efficacy may even surpass that of doxorubicin alone (58, 59). Retrospective clinical studies have also reported activity for regimens containing temozolomide (often combined with bevacizumab) or dacarbazine, with median PFS exceeding 6 months and some patients remaining progression-free at 12 months (60). Based on these preliminary results, a randomized phase II clinical trial (STRADA, NCT03023124) evaluating a first-line regimen of doxorubicin combined with dacarbazine is currently underway.

### 6.2 Novel cytotoxic agents

In addition to traditional chemotherapeutic agents, several novel cytotoxic drugs have demonstrated therapeutic potential. Trabectedin, an alkaloid derived from marine organisms, is approved for the treatment of previously treated advanced soft tissue sarcoma. This agent is effective in certain sarcoma subtypes and also exhibits activity against translocation-related sarcomas, such as SFT (61). Its mechanism of action may involve modulating the abnormal transcriptional processes driven by oncogenic fusion proteins (e.g., NAB2-STAT6) (62). Preclinical studies have confirmed its anti-tumor activity in SFT patient-derived xenograft models (59). Multiple retrospective clinical studies have reported clinical responses to trabectedin in SFT, although the reported PFS varies widely (2.3–11.6 months) (63, 64). Currently, the STRADA clinical trial includes a trabectedin treatment arm, which aims to provide prospective evidence for its efficacy.Eribulin, a microtubule inhibitor, is approved as second-line therapy for liposarcoma (65). In preclinical SFT models, eribulin has also demonstrated anti-tumor activity. Furthermore, sporadic case reports from phase II clinical trials in advanced soft tissue sarcoma suggest potential efficacy in patients with SFT (66, 67).

### 6.3 Overall role and challenges of chemotherapy

According to current evidence, systemic chemotherapy retains therapeutic value for advanced SFT, particularly in dedifferentiated subtypes. Anthracyclines (e.g., doxorubicin) are generally considered the mainstay of first-line chemotherapy, while ifosfamide, dacarbazine, and trabectedin are often used as subsequent options. However, the use of chemotherapy is limited by significant toxicity(e.g., myelosuppression, severe mucositis, nausea, and vomiting). In addition, clinicians must be vigilant for anthracycline-associated cardiotoxicity, ifosfamide-induced encephalopathy or nephrotoxicity, and the risk of rhabdomyolysis with trabectedin. Therefore, the application of chemotherapy requires a rigorous assessment of the benefit-risk ratio and should be managed at experienced medical centers. In the future, results from prospective clinical trials such as STRADA will help to better define the role of chemotherapy in the systemic treatment of SFT and optimize strategies for drug selection and sequencing.

## 7 Anti-angiogenic therapy

Given the highly vascular nature of SFT and the strong association between its driver gene fusion (NAB2-STAT6) and angiogenesis markers, anti-angiogenic therapy has emerged as an important systemic treatment option for advanced SFT (68, 69).

### 7.1 Key agents and clinical evidence

Several multi-targeted tyrosine kinase inhibitors have demonstrated activity in this field, with efficacy typically evaluated using both Choi criteria (which assess changes in tumor density) and RECIST criteria. Among these agents, the combination of temozolomide and bevacizumab was highlighted in an early study that confirmed the potential of this therapeutic strategy (60). This retrospective study included 14 patients, and reported a partial response rate of 79% and a disease control rate of 93% according to the Choi criteria. In contrast, the RECIST criteria indicated a partial response rate of 14% and a stable disease rate of 86%. The median PFS was 9.7 months according to the Choi criteria, and 10.8 months by RECIST; the median OS was 24.3 months. However, these results should be interpreted with caution due to potential biases, as the cohort included some patients with low-grade SFT. Additionally, the 8–12 week interval between imaging assessments may have influenced the PFS estimation.

#### 7.1.1 Pazopanib

Pazopanib primarily targets VEGFR1-3, PDGFR, and KIT. Its activity in SFT was first reported in an early prospective single-institution study involving 13 SFT patients (70). Among the 11 evaluable patients, the objective response rate (ORR) by RECIST criteria was 9%, with a disease control rate (DCR) of 82%. By Choi criteria, the ORR was 46% and the DCR remained 82%. The median PFS was 4.7 months, and the median OS was 13.3 months. Notably, the study did not provide details regarding the pathological aggressiveness grading, which may partly explain why these efficacy data appear inferior to those of subsequent, more selective studies. The efficacy of pazopanib was further validated in the first prospective phase II trial (GEIS-32), which systematically evaluated different SFT subtypes. This trial divided patients into aggressive (including malignant and dedifferentiated subtypes) and low-aggressiveness (typical) SFT cohorts according to their degree of aggressiveness. Aggressive SFT Cohort: Thirty-six patients were enrolled from 16 centers in Italy, France, and Spain. Central review showed that, by Choi criteria, the ORR was 51% and the DCR was 77%; by RECIST criteria, the ORR was 6% and the DCR was 66%. Median PFS was 5.57 months, and the 2-year OS rate was 73% (71). Notably, early findings indicated that patients with the dedifferentiated subtype had very poor outcomes; subsequent protocol amendments therefore excluded these patients. Low-Aggressiveness SFT Cohort: Thirty-four patients were enrolled in this cohort. According to central review, the ORR and DCR by Choi criteria were 58% and 97%, respectively, while by RECIST criteria the ORR was 6% and the DCR was 94%. Median PFS was significantly longer than that of the aggressive cohort, at 9.8 and 11.2 months (depending on the criteria used), respectively (72).

The significant difference in efficacy between these two cohorts is primarily attributed to fundamental differences in their intrinsic biological behavior: low-aggressiveness SFTs tend to follow a relatively indolent course even when metastasized. This finding underscores the importance of distinguishing SFT subtypes in clinical practice to predict pazopanib efficacy. Although preliminary studies suggested that certain gene fusions (e.g., NAB2 exon 6–STAT6 exon 16/17) might correlate with an angiogenic phenotype (68), these differences alone do not fully account for the observed variations in treatment response. The results of the GEIS-32 trial established the role of pazopanib in the treatment of advanced SFT—particularly in non-dedifferentiated subtypes—with efficacy superior to that of conventional chemotherapy.

#### 7.1.2 Sunitinib

Sunitinib acts on targets such as VEGFR1-3, PDGFRβ, FLT3, KIT, and RET.A single-center retrospective study involving 35 SFT patients with poor prognosis confirmed its therapeutic value in this population (73). The study assessed 31 patients (20 with malignant SFT and 11 with dedifferentiated SFT) using RECIST criteria, and 29 patients (19 malignant, 10 dedifferentiated) using Choi criteria. According to RECIST, the partial response (PR) rate was 6.5% (2 patients), and the disease control rate (DCR; PR plus stable disease [SD]) was 60.5%. Using the more sensitive Choi criteria, which take into account changes in tumor density, the PR rate increased markedly to 48%, and the DCR was 65%. The median PFS was 6 months by RECIST and 7 months by Choi, with a median OS of 16 months.A key finding was that patients with dedifferentiated SFT (48% of the cohort) had significantly worse responses to sunitinib compared to those with malignant, non-dedifferentiated subtypes. Although the study’s limitations—including variability in assessment interval—should be noted, the population primarily comprised pretreated, high-grade, or dedifferentiated high-risk cases. Thus, the results strongly support the therapeutic activity of sunitinib in refractory SFT, in line with previous reports of its ability to induce long-term disease stabilization (74, 75).Notably, an exploratory study (IMMUNOSARC-1)evaluating the combination of sunitinib and the PD-1 inhibitor nivolumab reported a median PFS of 7.5 months in 7 patients with SFT, suggesting that the combination did not significantly improve upon the expected efficacy of sunitinib monotherapy (69).

#### 7.1.3 Axitinib

Axitinib primarily targets VEGFR1-3, PDGFRβ, and KIT. A single-center prospective trial evaluated its efficacy in 17 SFT patients: according to Choi criteria, the partial response rate was 41.2% (7 patients), and the disease control rate (DCR) was 76.5% (13 patients). Notably, among 9 patients who had previously received other anti-angiogenic agents, 4 (44.4%) still achieved a partial response, supporting the clinical strategy of switching to alternative anti-angiogenic agents upon disease progression (i.e., “rotational therapy”). Consistent with findings for pazopanib, this study also found that patients with dedifferentiated SFT did not respond to axitinib. Given its efficacy and relatively manageable safety profile, axitinib may be considered a treatment option for advanced SFT, particularly as rotational therapy following prior treatment failure (72). It is important to note that these agents, including pazopanib and sunitinib, are associated with class-related adverse events such as hypertension, proteinuria, and hand-foot skin reaction. Pazopanib, in particular, requires careful monitoring due to its potential for QT prolongation and risk of heart failure. Therefore, patients—especially those with underlying cardiovascular disease—should be thoroughly evaluated and closely monitored before and during treatment.

### 7.2 Treatment strategy and challenges

In summary, anti-angiogenic drugs are the cornerstone of systemic therapy for advanced, non-dedifferentiated SFT, offering superior disease control compared to traditional chemotherapy. The study by Martin-Broto et al. (76) further highlights the central role and expanding clinical applications of these agents in managing this disease. In clinical practice, first-line treatment with drugs such as pazopanib, followed by switching to other anti-angiogenic agents with different mechanisms (e.g., sunitinib, or axitinib) upon disease progression, has become an effective strategy for managing non-dedifferentiated SFT. By contrast, SFTs with dedifferentiated subtypes generally respond poorly to all anti-angiogenic drugs, and treatment in these patients should be approached with caution. Moreover, common adverse effects —including hypertension, proteinuria, hand-foot skin reaction, and hypothyroidism—require proactive monitoring and management throughout therapy. It is also essential to use assessment methods such as the Choi criteria to accurately evaluate the early efficacy of these agents. Future research should further clarify optimal treatment sequencing, explore strategies to overcome drug resistance (particularly in dedifferentiated subtypes), and assess the potential of these agents in combination with radiotherapy, immunotherapy, and other treatments.

## 8 Immunotherapy

The application of immunotherapy in SFT remains at the exploratory stage. However, the presence of the disease’s driver gene fusion (NAB2-STAT6) may create a unique tumor microenvironment and modulate T-cell mediated immune responses in SFT, making immunotherapy a promising new avenue for research (77). Currently, data on the efficacy of PD-1/PD-L1 inhibitors in SFT are extremely limited. An analysis of the immune microenvironment in 16 intracranial SFTs found that, while all tumors expressed PD-L1, the expression pattern correlated with prognosis: diffuse strong staining—particularly in the absence of CD8+ T-cell infiltration—was associated with a shorter time to treatment failure and a higher risk of extracranial metastasis (78). However, other studies have produced inconsistent results; for example, some reports have found generally low levels of PD-L1 expression in SFT (79, 80). A phase II trial of pembrolizumab in sarcomas included one patient with SFT who achieved a partial response (81), suggesting potential benefit in a subset of patients, but no definitive conclusions can be drawn from a single case. Overall, available evidence indicates considerable heterogeneity in the immune microenvironment of SFT, and the predictive value of PD-L1 as a sole biomarker remains unconfirmed.

Advances in understanding the molecular mechanisms of SFT are driving the development of novel therapeutic strategies, including approaches that target tumor-specific antigens or core signaling pathways. Preferentially Expressed Antigen in Melanoma (PRAME) is currently one of the most promising tumor-associated antigens. Studies show that approximately 58% of SFTs exhibit high PRAME protein expression, which may contribute to immune evasion, a reduced proportion of antigen-presenting cells, and increased expression of the anti-phagocytic marker CD47 (80). Furthermore, higher levels of PRAME RNA expression are correlated with poorer prognosis (71, 80). Given PRAME’s potential role in immune escape, it is considered an ideal target for adoptive cellular immunotherapies such as T-cell receptor-engineered T-cell (TCR-T) therapy.

Driver Pathway-Related Targets: Directly targeting the oncogenic network driven by the NAB2-STAT6 fusion protein represents a fundamental strategy. Targeting EGR1, a key node in the NAB2-STAT6 signaling pathway, is regarded as a promising direction for future SFT therapy. Although EGR1 was previously explored as a potential therapeutic target in prostate cancer (82), subsequent research did not advance. Growth factor signaling pathways regulated by EGR1, such as IGF2 and FGFR, also offer therapeutic potential (83). Drawing on treatment strategies from other fusion gene-driven tumors (e.g., Ewing sarcoma), the development of approaches that directly target the NAB2-STAT6 fusion transcript or protein represents a highly promising avenue for precision therapy (84). Given SFT’s sensitivity to radiotherapy, combining immune checkpoint inhibitors with (low-dose) radiotherapy may capitalize on the “*in situ* vaccine” effect of radiotherapy and generate synergistic anti-tumor immune responses (85). Theoretically, genomically unstable dedifferentiated SFTs may generate more neoantigens and therefor be more sensitive to immunotherapy—an intriguing hypothesis that awaits clinical validation (86).

## 9 Prognostic factors, adjuvant treatment decision-making, and long-term follow-up

The clinical course of CNS SFT is unpredictable, and treatment decisions should be guided by an individualized assessment of recurrence risk. Although there is no universally accepted standard for prognostic factors informing adjuvant therapy, existing studies have identified several key indicators associated with an increased risk of recurrence, these can be categorized as shown in **Table 3** (87–89):

**Table 3:** Risk factors for recurrence and adjuvant treatment recommendations for CNS SFT.

Risk category	Specific indicators	Association with recurrence risk	Adjuvant treatment recommendations
Surgical Resection	Subtotal resection/biopsy vs. gross-total resection	Subtotal resection or biopsy is a strong predictor of local recurrence, with risk significantly higher than gross-total resection	For patients undergoing subtotal resection, even for WHO grade I tumors, postoperative radiotherapy is recommended to improve local control
Tumor Pathological Characteristics	High WHO grade (II/III), high mitotic count (>4/10 HPF), tumor necrosis	These are clear indicators of poor prognosis and are directly associated with an increased risk of recurrence	Postoperative adjuvant radiotherapy should be strongly considered, regardless of the extent of resection
Tumor Burden and Location	Tumor diameter (e.g., >5–6 cm), intracranial locations where gross-total resection is challenging	Larger tumor size and Larger tumor size and complex locations (e.g., skull base) are risk factors for recurrence	Assessment should be integrated with other risk factors; adjuvant treatment is generally recommended for high-risk individuals

Low-grade and completely resected tumors have a relatively low risk of recurrence; observation may be appropriate, but the individual’s risk should be thoroughly discussed with the patient.

Patients who possess any of the above high-risk factors should be informed of their relatively increased risk of recurrence and should participate in shared decision-making regarding the benefits and risks of postoperative adjuvant therapy. All patients with CNS SFT require long-term, regular postoperative follow-up. High-resolution MRI of the tumor site is recommended every six months for the first two to three years after surgery. If the condition remains stable, the interval can be gradually extended to annual scans, with lifelong surveillance advised. Regular imaging is essential for the early detection of recurrence or metastasis and allows for timely intervention.An integrated algorithm incorporating surgical extent, risk factors, radiotherapy indications, and systemic therapy options is presented in **Figure 1**.

## 10 Discussion

In summary, CNS SFT poses significant therapeutic challenges, primarily due to the difficulty of achieving gross-total resection in complex anatomical locations and the inherent tendency of these tumors to recur. Successful management of this disease depends heavily on a multidisciplinary team—including neurosurgeons, pathologists, radiologists, radiation oncologists, and medical oncologists—to develop individualized, comprehensive treatment strategies throughout the disease course.

Radical surgical resection remains the cornerstone of treatment, both for initial therapy and for managing recurrent or metastatic lesions. Surgical decision-making requires a careful balance between achieving maximal safe resection and preserving neurological function and quality of life. A long-term, systematic follow-up protocol should be established postoperatively. Clinical evaluation and MRI of the lesion site are recommended every six months for the first two to three years after surgery, transitioning to annual monitoring if the disease remains stable, with lifelong follow-up advised. For patients at high risk of recurrence, postoperative adjuvant therapy should be actively considered to improve prognosis. In cases of high-grade tumors (WHO grades II–III), or tumors of any grade that could not be completely resected, postoperative adjuvant radiotherapy is central to reducing the risk of local recurrence. For advanced, recurrent, or metastatic disease, anti-angiogenic targeted therapies (e.g., pazopanib, sunitinib) have demonstrated greater efficacy than traditional chemotherapy and now play an important role in management.Chemotherapy and immunotherapy are reserved as adjunctive or exploratory options in selected cases.

Looking ahead, given the rarity of SFT, fostering international, and multicenter collaboration is crucial. Future research should focus on conducting large-scale, prospective clinical trials to establish high-level evidence; deepening the understanding of downstream signaling pathways related to the NAB2-STAT6 fusion gene to identify novel therapeutic targets; and optimizing multimodal combination strategies—including targeted therapy, immunotherapy, and radiotherapy. Through continued basic and clinical research, there is hope to further improve the long-term prognosis for patients with this rare disease.As illustrated in **Figure 1**, the management of CNS SFT requires a multidisciplinary, risk−adapted approach.

## References

[1] Klemperer P Coleman BR . Primary neoplasms of the pleura. A report of five cases. Am J Ind Med. (1992) 22:1–31. doi: 10.1002/ajim.4700220103 1415270

[2] Louis DN Perry A Reifenberger G von Deimling A Figarella-Branger D Cavenee WK . The 2016 World Health Organization classification of tumors of the central nervous system: a summary. Acta Neuropathol. (2016) 131:803–20. doi: 10.1007/s00401-016-1545-1 27157931

[3] Li XL Fu WW Zhang S Chen DY Chen YP Wu J . Solitary fibrous tumor/hemangiopericytoma of central nervous system: a clinicopathologic analysis of 71 cases. Zhonghua Bing Li Xue Za Zhi. (2017) 46:465–70. doi: 10.3760/cma.j.issn.0529-5807.2017.07.004 28728219

[4] Klekamp J . Spinal ependymomas. Part 1: Intramedullary ependymomas. Neurosurgical Focus. (2015) 39:E6. doi: 10.3171/2015.5.FOCUS15161 26235023

[5] Colamaria A Carbone F Sacco M Corsi F Leone A Parbonetti G . Solitary fibrous tumor/hemangiopericytoma of the cervical spine: a systematic review of the literature with an illustrative case. Surg Neurol Int. (2022) 13:532. doi: 10.25259/SNI_722_2022 36447863 PMC9699857

[6] Apra C Mokhtari K Cornu P Peyre M Kalamarides M . Intracranial solitary fibrous tumors/hemangiopericytomas: first report of Malignant progression. J Neurosurg. (2018) 128:1719–24. doi: 10.3171/2017.1.JNS162593 28644098

[7] Wang J Zhao K Han L Jiao L Liu W Xu Y . Solitary fibrous tumor/hemangiopericytoma of spinal cord: a retrospective single-center study of 16 cases. World Neurosurg. (2019) 123:e629–629e638. doi: 10.1016/j.wneu.2018.12.004 30554000

[8] Apra C El Arbi A Montero AS Parker F Knafo S . Spinal solitary fibrous tumors: an original multicenter series and systematic review of presentation, management, and prognosis. Cancers (Basel). (2022) 14(12):2839. doi: 10.3390/cancers14122839 35740510 PMC9221085

[9] Gou Q Xie Y Ai P . Intracranial solitary fibrous tumor/hemangiopericytoma: role and choice of postoperative radiotherapy techniques. Front Oncol. (2022) 12:994335. doi: 10.3389/fonc.2022.994335 36249022 PMC9554559

[10] Wang L Yu J Shu D Huang B Wang Y Zhang L . Primary endodermal hemangiopericytoma/solitary fibrous tumor of the cervical spine: a case report and literature review. BMC Surg. (2021) 21:405. doi: 10.1186/s12893-021-01399-6 34837986 PMC8626743

[11] Chen Y Xu Z Liu M Xu H . Recurrent solitary fibrous tumor of the spinal cord: a case report and literature review. Clin Neuropathol. (2020) 39:86–91. doi: 10.5414/NP301192 31670648

[12] Jia Q Zhou Z Zhang D Yang J Liu C Wang T . Surgical management of spinal solitary fibrous tumor/hemangiopericytoma: a case series of 20 patients. Eur Spine J. (2018) 27:891–901. doi: 10.1007/s00586-017-5376-0 29127512

[13] Yi X Xiao D He Y Yin H Gong G Long X . Spinal solitary fibrous tumor/hemangiopericytoma: a clinicopathologic and radiologic analysis of eleven cases. World Neurosurg. (2017) 104:318–29. doi: 10.1016/j.wneu.2017.05.016 28512044

[14] Flores-Justa A López-García E García-Allut A Reyes-Santías RM . Solitary fibrous tumour/haemangiopericytoma of the spinal cord. Neurocirugia (Astur Engl Ed). (2018) 29:309–13. doi: 10.1016/j.neucir.2018.01.005 29559217

[15] Nagano A Ohno T Nishimoto Y Oshima K Shimizu K . Malignant solitary fibrous tumor of the lumbar spinal root mimicking schwannoma: a case report. Spine J. (2014) 14:e17–20. doi: 10.1016/j.spinee.2013.07.463 24120147

[16] Kim BJ Yoo E Choi J Kim S . Intradural-extramedullary solitary fibrous tumor of the thoracic spine: a case report. Radiol Case Rep. (2020) 15:709–11. doi: 10.1016/j.radcr.2020.02.038 32280404 PMC7139141

[17] Louis DN Perry A Wesseling P Brat DJ Cree IA Figarella-Branger D . The 2021 WHO classification of tumors of the central nervous system: a summary. Neuro Oncol. (2021) 23:1231–51. doi: 10.1093/neuonc/noab106 34185076 PMC8328013

[18] Kinslow CJ Bruce SS Rae AI Sheth SA McKhann GM Sisti MB . Solitary-fibrous tumor/hemangiopericytoma of the central nervous system: a population-based study. J Neuro-Oncol. (2018) 138:173–82. doi: 10.1007/s11060-018-2787-7 29427152

[19] Ghose A Guha G Kundu R Tew J Chaudhary R . CNS hemangiopericytoma: a systematic review of 523 patients. Am J Clin Oncol. (2017) 40:223–7. doi: 10.1097/COC.0000000000000146 25350465

[20] Casali PG Blay JY Abecassis N Bajpai J Bauer S Biagini R . Gastrointestinal stromal tumours: ESMO-EURACAN-GENTURIS clinical practice guidelines for diagnosis, treatment and follow-up. Ann Oncol. (2022) 33:20–33. doi: 10.1016/j.annonc.2021.09.005 34560242

[21] de Bernardi A Dufresne A Mishellany F Blay JY Ray-Coquard I Brahmi M . Novel therapeutic options for solitary fibrous tumor: anti-angiogenic therapy and beyond. Cancers (Basel). (2022) 14(4):1064. doi: 10.3390/cancers14041064 35205812 PMC8870479

[22] Carneiro SS Scheithauer BW Nascimento AG Hirose T Davis DH . Solitary fibrous tumor of the meninges: a lesion distinct from fibrous meningioma. A clinicopathologic and immunohistochemical study. Am J Clin Pathol. (1996) 106:217–24. doi: 10.1093/ajcp/106.2.217 8712177

[23] Kim JM Choi YL Kim YJ Park HK . Comparison and evaluation of risk factors for meningeal, pleural, and extrapleural solitary fibrous tumors: a clinicopathological study of 92 cases confirmed by STAT6 immunohistochemical staining. Pathol Res Pract. (2017) 213:619–25. doi: 10.1016/j.prp.2017.04.026 28552537

[24] Haas RL Walraven I Lecointe-Artzner E van Houdt WJ Scholten AN Strauss D . Management of meningeal solitary fibrous tumors/hemangiopericytoma; surgery alone or surgery plus postoperative radiotherapy. Acta Oncol. (2021) 60:35–41. doi: 10.1080/0284186X.2020.1826574 32988268

[25] Haas RL Walraven I Lecointe-Artzner E van Houdt WJ Strauss D Schrage Y . Extrameningeal solitary fibrous tumors-surgery alone or surgery plus perioperative radiotherapy: a retrospective study from the global solitary fibrous tumor initiative in collaboration with the Sarcoma Patients EuroNet. Cancer. (2020) 126:3002–12. doi: 10.1002/cncr.32911 32315454 PMC7318349

[26] Na MK Choi KS Lim TH Shin H Lee J Lee H . A systematic review and meta-analysis on the efficacy of postoperative radiotherapy after gross total resection of intracranial solitary fibrous tumors. Sci Rep. (2025) 15:23368. doi: 10.1038/s41598-025-02170-0 40603922 PMC12222894

[27] Alshwayyat S Kamal H Alshwayyat TA Alshwayyat M Alkhatib M Erjan A . Does adjuvant radiotherapy enhance survival in intracranial solitary fibrous tumor patients? World Neurosurg. (2025) 194:123545. doi: 10.1016/j.wneu.2024.12.004 39647524

[28] Cohen-Inbar O Lee CC Mousavi SH Kano H Mathieu D Meola A . Stereotactic radiosurgery for intracranial hemangiopericytomas: a multicenter study. J Neurosurg. (2017) 126:744–54. doi: 10.3171/2016.1.JNS152860 27104850

[29] Sung KS Moon JH Kim EH Kang SG Kim SH Suh C . Solitary fibrous tumor/hemangiopericytoma: treatment results based on the 2016 WHO classification. J Neurosurg. (2018) 130:418–25. doi: 10.3171/2017.9.JNS171057 29521591

[30] Piscopo AJ Chowdhury AJ Teferi N Lee S Challa M Petronek M . Surgical management of craniospinal axis solitary fibrous tumors: a single-institution case series and comprehensive review of the literature. Neurosurgery. (2024) 94:358–68. doi: 10.1227/neu.0000000000002692 37747216 PMC12245270

[31] Lee JH Jeon SH Park CK Park SH Yoon HI Chang JH . The role of postoperative radiotherapy in intracranial solitary fibrous tumor/hemangiopericytoma: a multi-institutional retrospective study (KROG 18-11). Cancer Res Treat. (2022) 54:65–74. doi: 10.4143/crt.2021.142 33781051 PMC8756112

[32] Krengli M Cena T Zilli T Jereczek-Fossa BA De Bari B Villa Freixa S . Radiotherapy in the treatment of extracranial hemangiopericytoma/solitary fibrous tumor: study from the Rare Cancer Network. Radiother Oncol. (2020) 144:114–20. doi: 10.1016/j.radonc.2019.11.011 31805515

[33] Haas RL Walraven I Lecointe-Artzner E Scholten AN van Houdt WJ Griffin AM . Radiation therapy as sole management for solitary fibrous tumors (SFT): a retrospective study from the global SFT initiative in collaboration with the Sarcoma Patients EuroNet. Int J Radiat Oncol Biol Phys. (2018) 101:1226–33. doi: 10.1016/j.ijrobp.2018.04.024 29859795

[34] Ton M Deng M Meixner E Eichkorn T Krämer A Seidensaal K . Efficacy and toxicity of photon, proton, and carbon ion radiotherapy in the treatment of intracranial solitary fibrous tumor/hemangiopericytoma. Radiat Oncol. (2024) 19:42. doi: 10.1186/s13014-024-02434-5 38553768 PMC10981281

[35] Combs SE Behnisch W Kulozik AE Huber PE Debus J Schulz-Ertner D . Intensity modulated radiotherapy (IMRT) and fractionated stereotactic radiotherapy (FSRT) for children with head-and-neck-rhabdomyosarcoma. BMC Cancer. (2007) 7:177. doi: 10.1186/1471-2407-7-177 17854490 PMC2077337

[36] Mjabber RE Chahid M Alami R Gouach HE Rami A Jaouad M . Successful multimodal management of central nervous system solitary fibrous tumor: a case report. Radiol Case Rep. (2025) 20:51–8. doi: 10.1016/j.radcr.2024.09.105 39429707 PMC11488408

[37] Rutkowski MJ Sughrue ME Kane AJ Aranda D Mills SA Barani IJ . Predictors of mortality following treatment of intracranial hemangiopericytoma. J Neurosurg. (2010) 113:333–9. doi: 10.3171/2010.3.JNS091882 20367074

[38] Thapa S Fujio S Kitazono I Yonenaga M Masuda K Kuroki S . Solitary fibrous tumor or hemangiopericytoma of the sella in an older patient treated with partial removal followed by fractionated gamma knife radiosurgery. NMC Case Rep J. (2021) 8:697–703. doi: 10.2176/nmccrj.cr.2021-0103 35079536 PMC8769461

[39] Mima M Demizu Y Jin D Hashimoto N Takagi M Terashima K . Particle therapy using carbon ions or protons as a definitive therapy for patients with primary sacral chordoma. Br J Radiol. (2014) 87:20130512. doi: 10.1259/bjr.20130512 24288399 PMC3898974

[40] Kong L Lu JJ . Reirradiation of locally recurrent nasopharyngeal cancer: history, advances, and promises for the future. Chin Clin Oncol. (2016) 5:26. doi: 10.21037/cco.2016.03.19 27121886

[41] Shiba S Okamoto M Kiyohara H Okazaki S Kaminuma T Shibuya K . Impact of carbon ion radiotherapy on inoperable bone sarcoma. Cancers (Basel). (2021) 13(5):1099. doi: 10.3390/cancers13051099 33806515 PMC7961536

[42] Murata K Endo K Aihara T Matsuoka Y Nishimura H Suzuki H . Salvage carbon ion radiotherapy for recurrent solitary fibrous tumor: a case report and literature review. J Orthop Surg (Hong Kong). (2020) 28:2309499019896099. doi: 10.1177/2309499019896099 32101079

[43] Shiba S Takakusagi Y Mizoguchi N Tsuchida K Shima S Kano K . Carbon-ion radiotherapy for inoperable solitary fibrous tumor of the skull base: a case report. In Vivo. (2023) 37:908–11. doi: 10.21873/invivo.13161 36881080 PMC10026639

[44] Tomomatsu Y Takasawa E Shiba S Okamoto M Ikota H Inomata K . Separation surgery and adjuvant carbon ion radiotherapy for a recurrent solitary fibrous tumor/hemangiopericytoma: a case report. Spine Surg Relat Res. (2023) 7:402–5. doi: 10.22603/ssrr.2022-0177 37636140 PMC10447192

[45] Petr J Platzek I Hofheinz F Mutsaerts H Asllani I van Osch M . Photon vs. proton radiochemotherapy: effects on brain tissue volume and perfusion. Radiother Oncol. (2018) 128:121–7. doi: 10.1016/j.radonc.2017.11.033 29370984

[46] Jones B McMahon SJ Prise KM . The radiobiology of proton therapy: Challenges and opportunities around relative biological effectiveness. Clin Oncol (R Coll Radiol). (2018) 30:285–92. doi: 10.1016/j.clon.2018.01.010 29454504

[47] Mohan R Grosshans D . Proton therapy - present and future. Adv Drug Delivery Rev. (2017) 109:26–44. doi: 10.1016/j.addr.2016.11.006 27919760 PMC5303653

[48] Ma M Gong Y Tang X Deng P Qian J Hu X . Solitary fibrous tumor in the saddle area treated with neuroendoscopic surgery and proton therapy: A case report and literature review. Oncol Lett. (2023) 26:505. doi: 10.3892/ol.2023.14092 37920432 PMC10618926

[49] De Jesus O Carballo Cuello CM Fernández-de Thomas RJ Pastrana EA . Gamma Knife radiosurgery for a recurrent craniocervical junction solitary fibrous tumour. BMJ Case Rep. (2022) 15(9):e250566. doi: 10.1136/bcr-2022-250566 36113959 PMC9486226

[50] Hajikarimloo B Tos SM Mohammadzadeh I Habibi MA . Postoperative stereotactic radiosurgery for intracranial solitary fibrous tumors/hemangiopericytomas: A systematic review and meta-analysis. J Clin Neurosci. (2025) 137:111302. doi: 10.1016/j.jocn.2025.111302 40339242

[51] Piccardo AC Gurdschinski S Spieker S Renner C Czapiewski P Wösle M . Repeated radiation therapy of recurrent solitary fibrous tumors of the brain: A medical case history over 20 years. Adv Radiat Oncol. (2024) 9:101426. doi: 10.1016/j.adro.2023.101426 38435964 PMC10906171

[52] Rana N Kim E Jaboin J Attia A . The role of adjuvant radiation in the management of solitary fibrous tumors of the central nervous system: A national cancer database analysis of 155 patients. Cureus. (2018) 10:e2656. doi: 10.7759/cureus.2656 30042907 PMC6054364

[53] Bishop AJ Zagars GK Demicco EG Wang WL Feig BW Guadagnolo BA . Soft tissue solitary fibrous tumor: Combined surgery and radiation therapy results in excellent local control. Am J Clin Oncol. (2018) 41:81–5. doi: 10.1097/COC.0000000000000218 26270446

[54] Schuetze SM Bolejack V Choy E Ganjoo KN Staddon AP Chow WA . Phase 2 study of dasatinib in patients with alveolar soft part sarcoma, chondrosarcoma, chordoma, epithelioid sarcoma, or solitary fibrous tumor. Cancer. (2017) 123:90–7. doi: 10.1002/cncr.30379 27696380

[55] Outani H Kobayashi E Wasa J Saito M Takenaka S Hayakawa K . Clinical outcomes of patients with metastatic solitary fibrous tumors: A Japanese Musculoskeletal Oncology Group (JMOG) multiinstitutional study. Ann Surg Oncol. (2021) 28:3893–901. doi: 10.1245/s10434-020-09306-8 33146837

[56] Levard A Derbel O Méeus P Ranchère D Ray-Coquard I Blay JY . Outcome of patients with advanced solitary fibrous tumors: the Centre Léon Bérard experience. BMC Cancer. (2013) 13:109. doi: 10.1186/1471-2407-13-109 23496996 PMC3623626

[57] Stacchiotti S Libertini M Negri T Palassini E Gronchi A Fatigoni S . Response to chemotherapy of solitary fibrous tumour: a retrospective study. Eur J Cancer. (2013) 49:2376–83. doi: 10.1016/j.ejca.2013.03.017 23566418

[58] Stacchiotti S Tortoreto M Bozzi F Tamborini E Morosi C Messina A . Dacarbazine in solitary fibrous tumor: a case series analysis and preclinical evidence vis-a-vis temozolomide and anti-angiogenics. Clin Cancer Res. (2013) 19:5192–201. doi: 10.1158/1078-0432.CCR-13-0776 23888069

[59] Stacchiotti S Saponara M Frapolli R Tortoreto M Cominetti D Provenzano S . Patient-derived solitary fibrous tumour xenografts predict high sensitivity to doxorubicin/dacarbazine combination confirmed in the clinic and highlight the potential effectiveness of trabectedin or eribulin against this tumour. Eur J Cancer. (2017) 76:84–92. doi: 10.1016/j.ejca.2017.02.002 28284173

[60] Park MS Patel SR Ludwig JA Trent JC Conrad CA Lazar AJ . Activity of temozolomide and bevacizumab in the treatment of locally advanced, recurrent, and metastatic hemangiopericytoma and Malignant solitary fibrous tumor. Cancer. (2011) 117:4939–47. doi: 10.1002/cncr.26098 21480200 PMC3135685

[61] Kawai A Araki N Sugiura H Ueda T Yonemoto T Takahashi M . Trabectedin monotherapy after standard chemotherapy versus best supportive care in patients with advanced, translocation-related sarcoma: a randomised, open-label, phase 2 study. Lancet Oncol. (2015) 16:406–16. doi: 10.1016/S1470-2045(15)70098-7 25795406

[62] Grohar PJ Segars LE Yeung C Pommier Y D'Incalci M Mendoza A . Dual targeting of EWS-FLI1 activity and the associated DNA damage response with trabectedin and SN38 synergistically inhibits Ewing sarcoma cell growth. Clin Cancer Res. (2014) 20:1190–203. doi: 10.1158/1078-0432.CCR-13-0901 24277455 PMC5510643

[63] Khalifa J Ouali M Chaltiel L Le Guellec S Le Cesne A Blay JY . Efficacy of trabectedin in Malignant solitary fibrous tumors: a retrospective analysis from the French Sarcoma Group. BMC Cancer. (2015) 15:700. doi: 10.1186/s12885-015-1697-8 26472661 PMC4608145

[64] Kobayashi H Iwata S Wakamatsu T Hayakawa K Yonemoto T Wasa J . Efficacy and safety of trabectedin for patients with unresectable and relapsed soft-tissue sarcoma in Japan: A Japanese Musculoskeletal Oncology Group study. Cancer. (2020) 126:1253–63. doi: 10.1002/cncr.32661 31825533

[65] Schöffski P Chawla S Maki RG Italiano A Gelderblom H Choy E . Eribulin versus dacarbazine in previously treated patients with advanced liposarcoma or leiomyosarcoma: a randomised, open-label, multicentre, phase 3 trial. Lancet. (2016) 387:1629–37. doi: 10.1016/S0140-6736(15)01283-0 26874885

[66] Schöffski P Ray-Coquard IL Cioffi A Bui NB Bauer S Hartmann JT . Activity of eribulin mesylate in patients with soft-tissue sarcoma: a phase 2 study in four independent histological subtypes. Lancet Oncol. (2011) 12:1045–52. doi: 10.1016/S1470-2045(11)70230-3 21937277

[67] Kawai A Araki N Naito Y Ozaki T Sugiura H Yazawa Y . Phase 2 study of eribulin in patients with previously treated advanced or metastatic soft tissue sarcoma. Jpn J Clin Oncol. (2017) 47:137–44. doi: 10.1093/jjco/hyw175 28173193 PMC5943671

[68] Bieg M Moskalev EA Will R Hebele S Schwarzbach M Schmeck S . Gene expression in solitary fibrous tumors (SFTs) correlates with anatomic localization and NAB2-STAT6 gene fusion variants. Am J Pathol. (2021) 191:602–17. doi: 10.1016/j.ajpath.2020.12.015 33497701

[69] Martin-Broto J Hindi N Grignani G Martinez-Trufero J Redondo A Valverde C . Nivolumab and sunitinib combination in advanced soft tissue sarcomas: a multicenter, single-arm, phase Ib/II trial. J Immunother Cancer. (2020) 8(2):e001561. doi: 10.1136/jitc-2020-001561 33203665 PMC7674086

[70] Maruzzo M Martin-Liberal J Messiou C Miah A Thway K Alvarado R . Pazopanib as first line treatment for solitary fibrous tumours: the Royal Marsden Hospital experience. Clin Sarcoma Res. (2015) 5:5. doi: 10.1186/s13569-015-0022-2 25664166 PMC4320530

[71] Martin-Broto J Stacchiotti S Lopez-Pousa A Redondo A Bernabeu D de Alava E . Pazopanib for treatment of advanced Malignant and dedifferentiated solitary fibrous tumour: a multicentre, single-arm, phase 2 trial. Lancet Oncol. (2019) 20:134–44. doi: 10.1016/S1470-2045(18)30676-4 30578023

[72] Martin-Broto J Cruz J Penel N Le Cesne A Hindi N Luna P . Pazopanib for treatment of typical solitary fibrous tumours: a multicentre, single-arm, phase 2 trial. Lancet Oncol. (2020) 21:456–66. doi: 10.1016/S1470-2045(19)30826-5 32066540

[73] Stacchiotti S Negri T Libertini M Palassini E Marrari A De Troia B . Sunitinib malate in solitary fibrous tumor (SFT). Ann Oncol. (2012) 23:3171–9. doi: 10.1093/annonc/mds143 22711763

[74] George S Merriam P Maki RG Van den Abbeele AD Yap JT Akhurst T . Multicenter phase II trial of sunitinib in the treatment of nongastrointestinal stromal tumor sarcomas. J Clin Oncol. (2009) 27:3154–60. doi: 10.1200/JCO.2008.20.9890 19451429 PMC2716937

[75] Domont J Massard C Lassau N Armand JP Le Cesne A Soria JC . Hemangiopericytoma and anti-angiogenic therapy: clinical benefit of anti-angiogenic therapy (sorafenib and sunitinib) in relapsed Malignant haemangioperyctoma /solitary fibrous tumour. Invest New Drugs. (2010) 28:199–202. doi: 10.1007/s10637-009-9249-1 19352594

[76] Martin-Broto J Mondaza-Hernandez JL Moura DS Hindi N . A comprehensive review on solitary fibrous tumor: New insights for new horizons. Cancers (Basel). (2021) 13(12):2913. doi: 10.3390/cancers13122913 34200924 PMC8230482

[77] Tazzari M Negri T Rini F Vergani B Huber V Villa A . Adaptive immune contexture at the tumour site and downmodulation of circulating myeloid-derived suppressor cells in the response of solitary fibrous tumour patients to anti-angiogenic therapy. Br J Cancer. (2014) 111:1350–62. doi: 10.1038/bjc.2014.437 25101565 PMC4183857

[78] Kamamoto D Ohara K Kitamura Y Yoshida K Kawakami Y Sasaki H . Association between programmed cell death ligand-1 expression and extracranial metastasis in intracranial solitary fibrous tumor/hemangiopericytoma. J Neuro-Oncol. (2018) 139:251–9. doi: 10.1007/s11060-018-2876-7 29675794

[79] Dancsok AR Setsu N Gao D Blay JY Thomas D Maki RG . Expression of lymphocyte immunoregulatory biomarkers in bone and soft-tissue sarcomas. Mod Pathol. (2019) 32:1772–85. doi: 10.1038/s41379-019-0312-y 31263176

[80] Wang WL Gokgoz N Samman B Andrulis IL Wunder JS Demicco EG . RNA expression profiling reveals PRAME, a potential immunotherapy target, is frequently expressed in solitary fibrous tumors. Mod Pathol. (2021) 34:951–60. doi: 10.1038/s41379-020-00687-5 33009490

[81] Toulmonde M Penel N Adam J Chevreau C Blay JY Le Cesne A . Use of PD-1 targeting, macrophage infiltration, and IDO pathway activation in sarcomas: A phase 2 clinical trial. JAMA Oncol. (2018) 4:93–7. doi: 10.1001/jamaoncol.2017.1617 28662235 PMC5833654

[82] Gitenay D Baron VT . Is EGR1 a potential target for prostate cancer therapy. Future Oncol. (2009) 5:993–1003. doi: 10.2217/fon.09.67 19792968 PMC2776080

[83] Huijbers E van der Werf IM Faber LD Sialino LD van der Laan P Holland HA . Targeting tumor vascular CD99 inhibits tumor growth. Front Immunol. (2019) 10:651. doi: 10.3389/fimmu.2019.00651 31001265 PMC6455290

[84] Ludwig JA Federman N Anderson P Macy ME Davis LE Riedel RF . 1620O phase I study of TK216, a novel anti-ETS agent for Ewing sarcoma. Ann Oncol. (2020) 31:S972. doi: 10.1016/j.annonc.2020.08.1846 38826717

[85] Martin-Broto J Hindi N Lopez-Pousa A Peinado-Serrano J Alvarez R Alvarez-Gonzalez A . Assessment of safety and efficacy of combined trabectedin and low-dose radiotherapy for patients with metastatic soft-tissue sarcomas: a nonrandomized phase 1/2 clinical trial. JAMA Oncol. (2020) 6:535–41. doi: 10.1001/jamaoncol.2019.6584 32077895 PMC7042948

[86] Chalmers ZR Connelly CF Fabrizio D Gay L Ali SM Ennis R . Analysis of 100,000 human cancer genomes reveals the landscape of tumor mutational burden. Genome Med. (2017) 9:34. doi: 10.1186/s13073-017-0424-2 28420421 PMC5395719

[87] Chen Q Chen XZ Wang JM Li SW Jiang T Dai JP . Intracranial meningeal hemangiopericytomas in children and adolescents: CT and MR imaging findings. AJNR Am J Neuroradiol. (2012) 33:195–9. doi: 10.3174/ajnr.A2721 22158928 PMC7966162

[88] Reisenauer JS Mneimneh W Jenkins S Mansfield AS Aubry MC Fritchie KJ . Comparison of risk stratification models to predict recurrence and survival in pleuropulmonary solitary fibrous tumor. J Thorac Oncol. (2018) 13:1349–62. doi: 10.1016/j.jtho.2018.05.040 29935303

[89] Demicco EG Wagner MJ Maki RG Gupta V Iofin I Lazar AJ . Risk assessment in solitary fibrous tumors: validation and refinement of a risk stratification model. Mod Pathol. (2017) 30:1433–42. doi: 10.1038/modpathol.2017.54 28731041

